# Disease scoring systems for oral lichen planus; a critical appraisal

**DOI:** 10.4317/medoral.20524

**Published:** 2015-02-14

**Authors:** Jing Wang, Isaäc van der Waal

**Affiliations:** 1 Department of Oral and Maxillofacial Surgery/Pathology, VU University medical center (VUmc) and Academic Centre for Dentistry Amsterdam (ACTA), Amsterdam, The Netherlands

## Abstract

The aim of the present study has been to critically review 22 disease scoring systems (DSSs) on oral lichen planus (OLP) that have been reported in the literature during the past decades. Although the presently available DSSs may all have some merit, particularly for research purposes, the diversity of both the objective and subjective parameters used in these systems and the lack of acceptance of one of these systems for uniform use, there is a need for an international, authorized consensus meeting on this subject. Because of the natural course of OLP characterized by remissions and exacerbations and also due to the varying distribution pattern and the varying clinical types, e.g. reticular and erosive, the relevance of a DSS based on morphologic parameters is somewhat questionable. Instead, one may consider to only look for a quality of life scoring system adapted for use in OLP patients.

** Key words:**Oral lichen planus, disease scoring system, classification.

## Introduction

Lichen planus is a chronic autoimmune, inflammatory like mucocutaneous disease. The exact etiopathogenesis is still unknown. The disease occurs more often in females than in males with a ratio of approximately 2:1 and mostly affects the middle-aged population. The reported prevalence of lichen planus in general is up to 5%, while the prevalence of oral lichen planus (OLP) is set at 1.2% (1).

Cutaneous lichen planus (CLP) includes skin and nails. The lesions are distributed in a bilateral and more or less symmetrical pattern (2). Mucosal lichen planus (MLP) may involve the mouth, gastrointestinal tract, larynx, genitals, ears, nose, bladder and conjunctivae of the eyes. Oral lichen planus may occur isolated or in combination with involvement of cutaneous sites or other mucosal sites. In the past, six clinical types of OLP have been recognized, being reticular, papular, plaquelike, atrophic, ulcerative/erosive, and bullous respectively (3). There is a trend nowadays to divide OLP into three categories, being 1) reticular/popular/plaque type, 2) erosive/erythematous type, and 3) ulcerative type (4). More than one subtype of OLP may occur in the same patient and subtypes may vary in the individual patients during the course of the disease. As in CLP, there is usually a bilateral and symmetrical distribution. Reticular OLP, which is probably the most common presentation, is usually asymptomatic while the other types may cause pain or discomfort, either spontaneously or during meals, e.g. during the use of spicy food. The clinical course of OLP is characterized by remissions and exacerbations. At present, there is no effective treatment for OLP (5). Therefore, treatment can only be symptomatic and usually consists of topical application of corticosteroids either as an ointment or a mouth rinse.

In the past decades, several disease scoring systems (DSSs) for OLP have been reported. The aim of the present study is to critically review these scoring systems.

## Material and Methods

A Pubmed and Google Scholar search has been performed using the keywords “(oral) lichen planus”, “scoring system”, and “classification” to retrieve publications on Disease Scoring Systems for oral lichen planus in the English literature since 1980.

## Results

As a result of the search a total number of 22 publications have been collected. A brief summary of the various parameters that have been used in the various DSSs is depicted in  table 1 and  table 1 continued. It is obvious that there is a wide variety in the objective and symptomatic parameters that have been used in the various DSSs, ranging from just the use of a VAS score in the study from Silverman *et al*. (10) to a rather detailed system in the studies from Pinboonniyom *et al*. (4) and Escudier *et al*. (20).

**Table 1:**
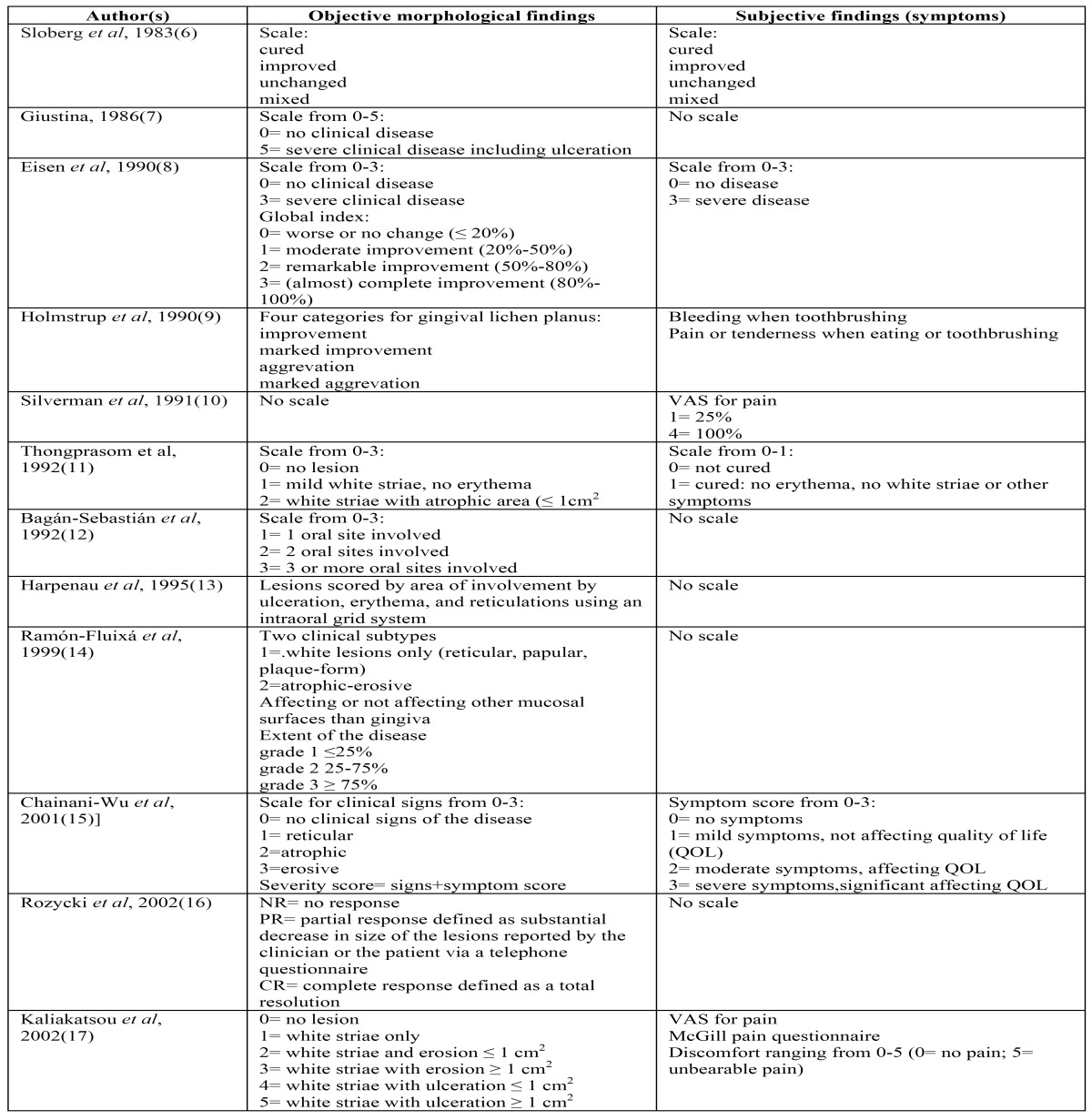
Reported Disease Scoring Systems for oral lichen planus (4,6-26).

**Table 1 Continued:**
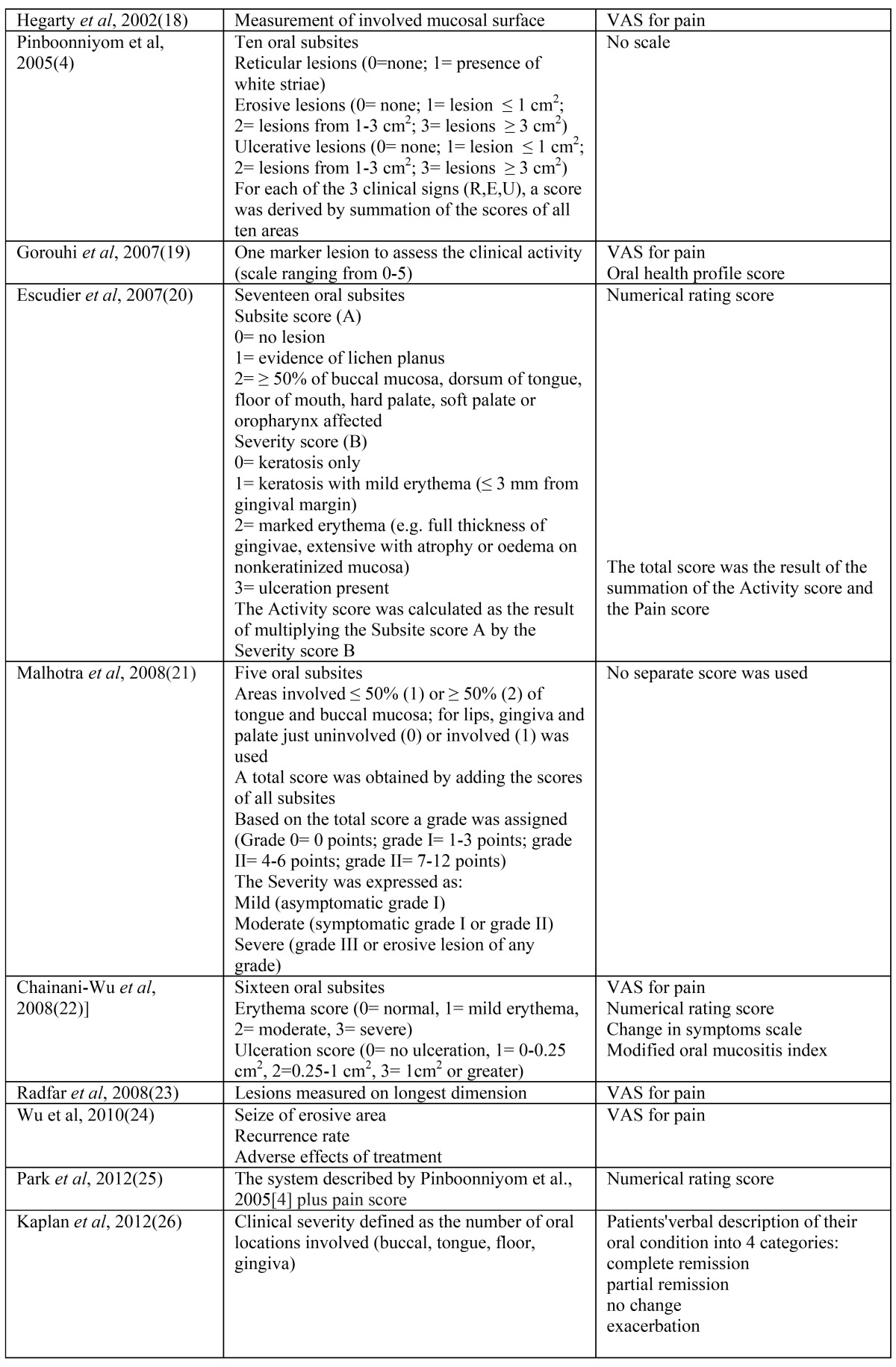
Reported Disease Scoring Systems for oral lichen planus (4,6-26).

## Discussion and Conclusions

The number of collected publications on DSSs for OLP is actually higher than twenty-two, since a number of scoring systems have been used in studies on the symptomatic treatment of OLP that did not focus on the disease scoring system, as is shown in a review in 2011 of treatment interventions (27). Nevertheless, the publications, as being listed in  Table 1, are representative of the various types of DSSs. Almost all of the reported DSSs are based on the extent of the disease, i.e. the number of affected oral subsites, the size of the lesions and the clinical presentation, such as reticular, erosive/erythematous or ulcerative with or without the use of scoring system for symptoms. In the various DSSs there are differences in the use of the terms activity, severity and extent of the disease. In some DSSs the erosive type of OLP is graded as a more severe type of OLP than the reticular type, while in such studies also a pain score has been applied. In yet other DSSs the extent of the disease is used as a parameter for the severity or the activity of OLP.

Remarkably, there is only one study in which two of the reported DSSs have been compared for their applicability (28); not surprisingly, the authors of that study recommended to design a uniform scoring system. Indeed, due to the diversity of both the objective and subjective parameters that have been used in the various DSSs it impossible to compare the results obtained from different studies on the treatment of oral lichen planus.

**Figure 1:**
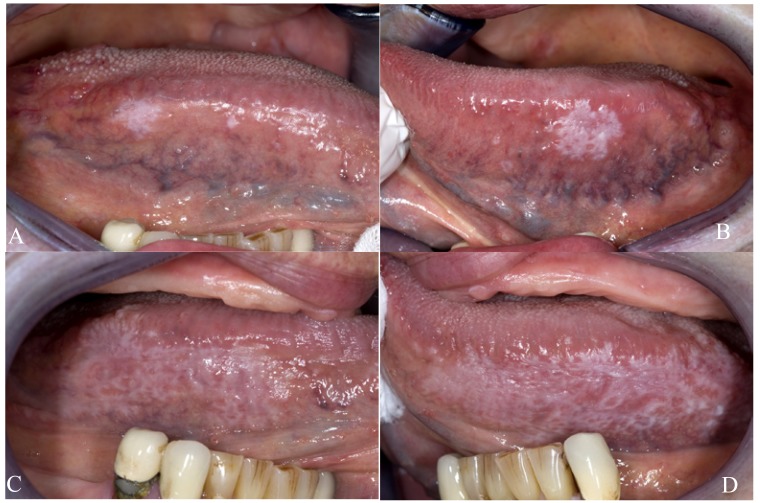
Plaque-type lichen planus on the borders of the tongue; mild symptoms (a,b); same patients after two months; symptoms unchanged as reported by the patient (c,d). How to incorporate the morphological changes in a disease scoring system? A quality of life scoring system may be more useful.

**Figure 2:**
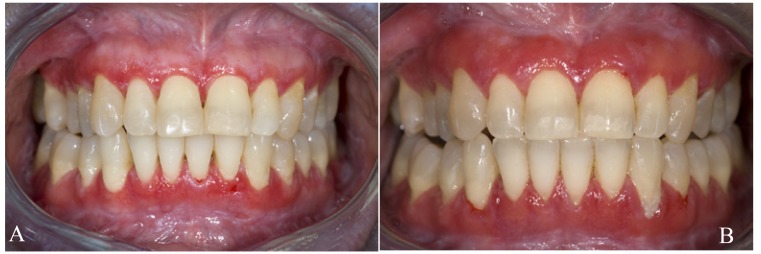
Erosive lichen planus in the upper and lower gingiva; mild symptoms, mainly consisting of bleeding on toothbrushing (a); same patient after six months; symptoms unchanged as reported by the patient (b). How to incorporate the morphological changes in a disease scoring system? A quality of life scoring system may be more useful.

Because of its natural course of remissions and exacerbations, sometimes lifelong, and also due to the varying distribution pattern and the varying clinical types, e.g. reticular and erosive (Figs. 1 and 2), the use of a DSS for the assessment of treatment results in OLP is rather questionable.

Silverman did not use objective, morphological parameters in his study but merely used a visual analog scale scale (VAS) for pain (10). With regard to gingival OLP one may question whether pain alone is a proper parameter. Patients with erosive OLP of the gingiva may not so much complain of pain but rather of bleeding on tooth brushing; in some of these patients the main complaint may even be an aesthetic one. In this respect, one may consider to develop a symptomatic scoring system for patients with OLP of the gingiva only. Yet other patients with OLP only report pain or discomfort when eating spicy food. Therefore, as has been mentioned already by several authors (15,19), the use of a quality of life scoring system (29), adjusted for use in OLP patients, may be considered, perhaps even as the only parameter for the assessment of treatment results.

In conclusion, the presently available disease scoring systems for oral lichen planus may all have some merit, particularly for research purposes. Because of the diversity of these systems and the lack of acceptance of one of these systems for uniform use, there is a need for an international, authorized consensus meeting on this subject. One actually may consider to only design a quality of life scoring system adapted for use in OLP patients.
